# Open-labeled, multicenter phase II study of prophylactic administration of pegylated granulocyte colony-stimulating factor in relapsed or refractory multiple myeloma who received pomalidomide-based regimens (KMM170)

**DOI:** 10.3389/fonc.2023.1209110

**Published:** 2023-10-25

**Authors:** Ga-Young Song, Je-Jung Lee, Joon Ho Moon, Dajung Kim, Min Kyoung Kim, Hyo Jung Kim, Yeung-Chul Mun, Won-Sik Lee, Young Rok Do, Jae Hoon Lee, Sung-Hoon Jung, Jin Seok Kim

**Affiliations:** ^1^ Department of Hemotology-Oncology, Chonnam National University Hwasun Hospital and Chonnam National University Medical School, Hwasun, Jeollanamdo, Republic of Korea; ^2^ Department of Hematology-Oncology, Kyungpook National University Hospital, School of Medicine, Kyungpook National University, Daegu, Republic of Korea; ^3^ Department of Internal Medicine, Kosin University Gospel Hospital, Busan, Republic of Korea; ^4^ Department of Hematology and Oncology, Yeungnam University Medical Center, Daegu, Republic of Korea; ^5^ Department of Internal Medicine, Hallym University Sacred Heart Hospital, Anyang, Republic of Korea; ^6^ Division of Hematology-Oncology, Ewha Womans University School of Medicine, Seoul, Republic of Korea; ^7^ Busan Paik Hospital, Inje University, Busan, Republic of Korea; ^8^ Department of Internal Medicine, Keimyung University, School of Medicine, Keimyung University Hospital, Daegu, Republic of Korea; ^9^ Department of Internal Medicine, Gachon University Gil Medical Center, Incheon, Republic of Korea; ^10^ Division of Hematology, Department of Internal Medicine, Severance Hospital, Yonsei University College of Medicine, Seoul, Republic of Korea

**Keywords:** pegfilgrastim, prophylaxis, febrile neutropenia, multiple myeloma, pomalidomide

## Abstract

**Introduction:**

Pegylated granulocyte colony-stimulating factor (G-CSF) has been widely used for preventing febrile neutropenia in various types of cancer treatment. In the present study, we prospectively evaluated the safety and efficacy of pegfilgrastim as a primary prophylaxis of febrile neutropenia and infection among patients with relapsed refractory multiple myeloma (RRMM) treated with pomalidomide-based regimens.

**Methods:**

Thirty-three patients with RRMM who received pomalidomide and dexamethasone (Pd) with or without cyclophosphamide (PCd) were enrolled in this study. Twenty-eight patients were treated with PCd and 5 patients were treated with Pd. All patients were given pegfilgrastim subcutaneously with a single administration performed on the first day of each cycle as primary prophylaxis until the fourth cycle.

**Results:**

The median age of the patients was 75 (range 56-85), and the median prior line of therapy was 2 (range 2-6). Seventeen patients (51.5%) had any grade of neutropenia and 20 (60.6%) had any grade of thrombocytopenia before starting pomalidomide treatment. During the 4 cycles of treatment, grade 3 or more neutropenia occurred in 17 patients (51.5%), and 4 (12.1%) experienced grade 3 or more febrile neutropenia. Grade 3 or more infections occurred in 5 patients (15.2%). Interestingly, the patients with markedly increased ANC of more than 2 x 109/L compared to baseline ANC after 7 days of pegfilgrastim at 1st cycle of treatment showed a significantly lower incidence of grade 3-4 neutropenia. The most common adverse event of pegfilgrastim was fatigue, and all the adverse events caused by pegfilgrastim were grade 1 or 2. And there was no significant change in the immune cell population and cytokines during the administration of pegfilgrastim.

**Discussion:**

Considering that this study included elderly patients with baseline neutropenia, pegylated G-CSF could be helpful to prevent severe neutropenia, febrile neutropenia, or infection in patients with RRMM.

## Introduction

Pomalidomide is a third-generation immunomodulatory drug (IMiD) that is pharmacologically distinct from lenalidomide and demonstrated efficacy in combination with dexamethasone in patients with relapsed refractory multiple myeloma (RRMM) (1). In the randomized phase 3 trial, pomalidomide in combination with low-dose dexamethasone (Pd) showed significantly better overall response rate (ORR) (31% vs 10%, *p* < 0.0001) and longer progression-free survival (PFS) (3.8 vs 1.9 months; HR 0.41, *p* < 0.0001) and overall survival (OS) (11.9 vs 7.8 months; HR 0.53, *p* = 0.0002) compared to high-dose dexamethasone alone. The most common toxicity in this study was grade 3-4 neutropenia, which occurred in 48% of patients treated with Pd. In addition, about 50% of patients received granulocyte colony-stimulating factor (G-CSF) for recovery of severe neutropenia. Recently, pomalidomide, cyclophosphamide, and dexamethasone (PCd) regimen are mainly used rather than Pd because the addition of weekly cyclophosphamide to Pd resulted in improved outcomes compared to Pd (2, 3). However, the addition of cyclophosphamide can worsen the incidence of severe neutropenia. In real clinical practice, grade 3-4 neutropenia and infection were reported to be 97.4% and 63.1% of the patients treated with PCd (4).

In general, G-CSF may be used to prevent the occurrence of severe neutropenia and febrile neutropenia, but the use of prophylactic G-CSF is limited due to a short half-life of 3-4 hours. Pegylated G-CSF was developed to extend the half-life of G-CSF by decreasing systemic clearance, and once injection has efficacy comparable to that of daily G-CSF injection (5, 6). Pegylated G-CSF has been currently used as a primary prophylaxis of febrile neutropenia (FN) in the treatment of solid tumors or malignant lymphoma (2, 7–9). Some observational studies reported the efficacy of pegylated G-CSF prophylaxis in MM, but there is a lack of prospective trials (10, 11). In the present study, we conducted a multicenter, prospective phase 2 study to evaluate the primary prophylactic effect of pegylated G-CSF for reducing the incidence of severe neutropenia and febrile neutropenia in patients with RRMM treated with Pd or PCd.

## Methods

### Patients

The present study is a multicenter, prospective phase 2 study, and inclusion criteria are as follows: Patients with RRMM who were treated with pomalidomide-containing regimens were included. Patients were aged more than 19 and had an Eastern Cooperative Oncology Group (ECOG) performance status (PS) score of 0-2. Patients with smoldering MM and plasma cell leukemia were excluded, and patients who received radiation therapy within 4 weeks of the screening date were also excluded. Patients with an end-stage renal disease requiring dialysis, uncontrolled infection, severely impaired liver function; aspartate aminotransferase (AST) or alanine aminotransferase (ALT) of more than 3 times of upper normal limit, or platelet (PLT) count less than 50 x 10^9^/L without transfusion were also excluded in this study. However, there was no exclusion criterion for absolute neutrophil count (ANC) at the time of enrollment. This study was approved by the Institutional Review Board of each participating institution and was registered at https://cris.nih.go.kr/ with the identification number KCT 0002985. All of the procedures were conducted in accordance with the principles of the Declaration of Helsinki and local law. All of the patients provided written informed consent prior to enrollment.

### Treatment protocol

Patients underwent a 28-day treatment cycle; pomalidomide (4 mg on days 1-21, orally) plus dexamethasone (40 mg on days 1,8,15, and 22, orally) or plus cyclophosphamide (400 mg/day on days 1,8,15, orally). The dose of pomalidomide was reduced to 3 mg/day in patients with thrombocytopenia at the time of starting pomalidomide, and the dose of dexamethasone was reduced to 20 mg/day in patients older than 75 years old. Subsequent dose reduction of pomalidomide, cyclophosphamide, or dexamethasone was determined by the investigator’s decision according to the toxicity. All patients received pegfilgrastim subcutaneously with a single administration performed on the first day of each cycle as primary prophylaxis until the fourth cycle, and dose modification of pegfilgrastim was not allowed. The additional use of short-acting G-CSF was allowed when ANC decreased to less than 1 x 10^9^/L during Pd or PCd treatment. Patients received aspirin at a dose of 100 mg/day for thromboprophylaxis. Prophylactic antibiotics including levofloxacin 500 mg once daily, acyclovir 400 mg once daily, and trimethoprim/sulfamethoxazole 480 mg once daily were administered according to the investigator’s discretion to prevent infection. Pd or PCd therapy was continued until progressive disease (PD) or unacceptable toxicity was observed.

Treatment response was assessed according to the International Myeloma Working Group uniform response criteria using serum and 24-hour urine protein electrophoresis and serum free light chain ratio. Complete blood count (CBC) with differential count and C-Reactive protein (CRP) were assessed on days 1 and 8 during the first cycle of pomalidomide treatment, and on days 1, 15 ± 7 during 2nd to fourth cycle. Hematologic and non-hematologic toxicity was assessed every cycle. For the immunologic response assessment, peripheral blood of the patients was obtained on day 1 of each cycle and dynamic changes of regulatory T lymphocytes (Tregs) and cytokines such as interferon-gamma (IFN-γ), IL12p70, and TGF-β were analyzed (**Supplementary method**).

### Study endpoints and definition

The primary endpoints of this study were the incidence of grade 3 or more neutropenia (ANC of less than 1 x 10^9^/L) and grade 3 or more febrile neutropenia. Febrile neutropenia was defined as fever, a single axillary temperature greater than 38.0°C or an axillary temperature greater than 37.5°C lasting 1 hour, and neutropenia of ANC less than 2 x 10^9^/L. Infection was defined as the existence of a pathogen or imaging evidence of infection combined with concomitant clinical symptoms. The secondary endpoints were the duration of severe neutropenia, the incidence and duration of hospitalization due to toxicity during treatment, the incidence of adverse events associated with pegfilgrastim, and immunologic changes according to the administration of pegfilgrastim. The revised international staging system (R-ISS) was used to assess the clinical stage at diagnosis. Cytogenetic risk at diagnosis was classified into high-risk and standard-risk based on conventional cytogenetic studies or fluorescent *in situ* hybridization. Patients with del(17p), t(4,14), t(14,16), or amp(1q) were defined as having high-risk cytogenetic abnormalities. Adverse events were graded according to National Cancer Institute Common Terminology Criteria for Adverse Events (NCI-CTCAE) version 5.0.

### Statistical analysis

PFS was defined as the period from the date of starting pomalidomide-based regimens to the date of disease progression or death from any cause. OS was defined as the period from the date of starting pomalidomide-based regimens to the date of the last follow-up or death from any cause. PFS and OS were investigated using the Kaplan-Meier method and compared using the log-rank test. A *p*-value less than 0.05 was considered statistically significant. All the statistical analyses were performed using SPSS software (ver. 21; SPSS Inc., Chicago, IL, USA).

## Results

### Patient characteristics and clinical outcome

Thirty-three patients with RRMM who received Pd or PCd between March 2018 and September 2021 at 7 institutions in Korea were included in this study. Twenty-eight patients (84.8%) were treated with PCd and 5 (15.2%) were treated with Pd. The median age of the patients at the time of beginning the pomalidomide–based regimen was 75 (range 56-85). Nine patients (27.3%) had a high-risk cytogenetic abnormality at diagnosis, and 2 patients (6.1%) were categorized as R-ISS I, 20 (60.6%) were R-ISS II, and 5 (15.2%) were R-ISS III. The median duration from diagnosis to starting pomalidomide treatment was 33.0 months (range 6.3-128.2 months). The median prior line of therapy was 2 (range 2-6). Regarding antibiotic prophylaxis, 23 patients (69.7%) received levofloxacin, 9 patients (27.3%) received trimethoprim-sulfamethoxazole, and 21 patients (63.6%) received acyclovir. Seventeen patients (51.5%) already had any grade of neutropenia at the time of initiation of pomalidomide-based treatments, and 4 patients (12.1%) had more than grade 3 of neutropenia. Twenty patients (60.6%) had grade 1 or 2 thrombocytopenia at the time of pomalidomide treatment. The baseline clinical characteristics of the patients were summarized in **Table 1**.

**Table 1:** Baseline clinical characteristics of 33 patients before pomalidomide treatment.

Characteristics	Total (N=33)	PCd (n=28)	Pd (n=5)
Age, median (range)	75 (56–85)	75 (56–85)	79 (66–83)
Male, n (%)	18 (54.5)	15 (53.6)	3 (60.0)
ECOG PS, n (%)			
0-1 ≥2	28 (84.8) 5 (15.2)	24 (85.7) 4 (14.3)	4 (80.0) 1 (20.0)
Immunoglobulin heavy chain, n (%)			
IgG IgA Light chain	19 (57.6) 9 (27.3) 5 (15.2)	15 (53.6) 8 (28.6) 5 (17.9)	4 (80.0) 1 (20.0) 0 (0.0)
R-ISS at diagnosis, n (%)			
I II III	2 (6.1) 20 (60.6) 5 (15.2)	1 (3.6) 18 (64.3) 4 (14.3)	1 (20.0) 2 (40.0) 1 (20.0)
High-risk cytogenetics at diagnosis, n (%)	9 (27.3)	6 (21.4)	3 (75.0)
Beta2 microglobulin (mg/L), median (range)	6.44 (1.60-13.67)	6.26 (0.16-13.2)	8.32 (2.41-13.67)
WBC, x10^9^/L, median (range)	4.2 (7.1-11.8)	4.3 (7.1-11.8)	3.2 (2.7-6.1)
ANC, x10^9^/L, median (range)	1.9 (0.5-7.8)	1.9 (0.5-7.8)	1.9 (0.9-3.3)
Hb (/dL), median (range)	10.1 (7.9-13.5)	10.0 (7.9-13.5)	10.1 (8.1-11.0)
PLT, x10^9^/L, median (range)	120 (51–301)	120 (51–301)	110 (54–194)

N, number; ECOG, Eastern Cooperative Oncology Group; PS, performance status; Ig, immunoglobulin; R-ISS, Revised International Staging System; WBC, white blood cell count; ANC, absolute neutrophil count; Hb, hemoglobin; PLT, platelet.

### Incidence rates of neutropenia and infection

Clinical events associated with neutropenia are described in **Table 2**. During the four cycles of treatment, any grade of neutropenia occurred in 19 patients (57.6%); 16 (57.1%) in PCd, and 3 (60.0%) in Pd. Grade 3 or more neutropenia occurred in 17 patients (51.5%); 15 (53.6%) in PCd, and 2 (40.0%) in Pd. The median duration of grade 3 or higher neutropenia was 10 days (range 1-34). Four patients (12.1%) experienced grade 3 or more febrile neutropenia; 3 in PCd, and 1 in Pd. Conventional short-acting G-CSF was administrated when grade 3 or 4 neutropenia occurred. Total 17 patients experienced grade 3 or 4 neutropenia and subsequently received short-acting G-CSF during the study.

**Table 2:** Clinical events during the 4 cycles of pomalidomide treatment.

Event	Total (n=33)	PCd (n=28)	Pd (n=5)
Adverse event			
Overall (Gr1-4) neutropenia, n (%)	19 (57.6)	16 (57.1)	3 (60.0)
Gr3 neutropenia, n (%) Gr4 neutropenia, n (%)	7 (21.2) 10 (30.3)	6 (21.4) 9 (32.1)	1 (20.0) 1 (20.0)
Duration of Gr3-4 neutropenia (days), median (range)	10 (1-34)	10 (1-34)	7 (4-28)
Overall (Gr1-4) Febrile neutropenia, n (%) Gr3 Febrile neutropenia, n (%) Gr4 Febrile neutropenia, n (%)	4 (12.1) 1 (3.0) 3 (9.1)	3 (10.7) 1 (3.6) 2 (7.1)	1 (20.0) 0 (0.0) 1 (20.0)
Overall (Gr1-4) Infection, n (%) Gr3 infection, n (%) Gr4 infection, n (%)	7 (21.2) 3 (9.1) 1 (3.0)	7 (25.0) 3 (10.7) 1 (3.6)	0 (0.0) 0 (0.0) 0 (0.0)
Dose adjustment (dose reduction or treatment interruption)			
Pomalidomide, n (%)	12 (36.4)	11 (39.3)	1 (20.0)
Cyclophosphamide, n (%)	12 (36.4)	12 (42.9)	0 (0.0)
Dexamethasone, n (%)	9 (27.3)	8 (28.6)	1 (20.0)
Treatment delay > 2days, n (%)	17 (51.5)	14 (50.0)	3 (60.0)
Total dose of conventional G-CSF (mcg/kg), median (range)	8.5 (2.0-1652.0)	5.0 (3.0-45.0)	12.0 (2.0-1652.0)

**Figure 1:**
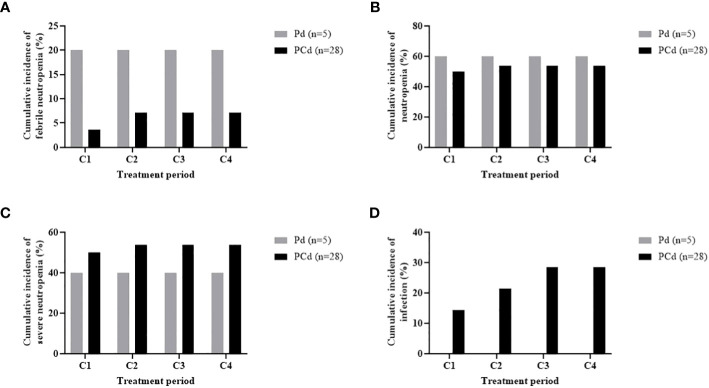
Cumulative incidence of any grade of febrile neutropenia **(A)**, any grade of neutropenia **(B)**, grade 3-4 neutropenia **(C)**, and any grade of infection **(D)** according to treatment period.

**Figure 2:**
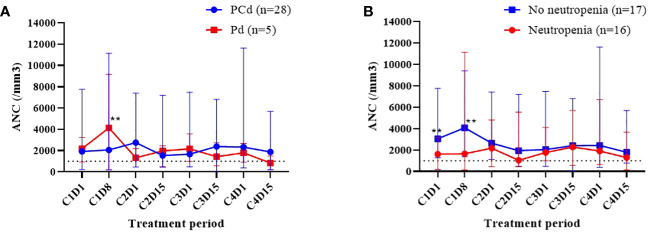
Dynamic change of white blood cell counts (WBC) during the treatment. Comparison of absolute neutrophil count (ANC) according to the treatment regimen **(A)**, the existence of any grade of neutropenia at the time of initiation of pomalidomide treatment **(B)**. **The blue dot and red dot values show significant difference (p<0.05).

N, number; Gr, grade;

The cumulative incidences of any grade of neutropenia, grade 3 or more neutropenia, any grade of febrile neutropenia, and infection during each regimen cycle are described in **Figure 1**, and the dynamic change of ANC during the treatment is shown in **Figure 2**. Changes in ANC showed no significant difference according to the treatment regimen (**Figure 2A**), and ANC was lower in those patients than in patients with baseline neutropenia of ANC less than 2 x 10^9^/L (**Figure 2B**). In 17 patients whose baseline ANC was less than 2 x 10^9^/L, the median ANC increase tended to be lower although statistically not significant (1.10 x 10^9^/L, range 0.07-7.99 x 10^9^/L vs. 2.44 x 10^9^/L, range 0.14-6.53 x 10^9^/L, *p*=0.345). Patients with baseline neutropenia and patients without baseline neutropenia experienced grade 3 or 4 neutropenia and grade 3 or 4 febrile neutropenia as follows (grade 3 or 4 neutropenia, 58.8% in patients with baseline neutropenia vs. 43.8% in patients without baseline neutropenia, *p*=0.494; grade 3 or 4 febrile neutropenia, 17.6% in patients with baseline neutropenia vs. 6.3% in patients without baseline neutropenia, *p*=0.601) during treatment (**Supplementary Table 1**). Nine patients showed markedly increased ANC of more than 2 x 10^9^/L compared to baseline ANC after 7 days of pegfilgrastim at 1st cycle of treatment, and the incidence of neutropenia was significantly lower in these 9 patients with markedly increased ANC after pegfilgrastim injection (Grade 1-4 neutropenia, 11.1% vs. 75.0%, *p*=0.002; Grade 3-4 neutropenia, 11.1% vs. 66.7%, *p*=0.007). No febrile neutropenia was developed in these 9 patients (**Table 3**). Any grade of infection occurred in 7 patients (21.2%) and grade 3 or more infection occurred in 4 patients (12.1%), and 5 of 7 infections occurred during the neutropenic period (3 of 4 grade 3 or more infections). The cause of infection was identified in 3 of the 7 patients; 2 were pneumonia, and 1 was urinary tract infection. Six patients recovered with broad-spectrum antibiotics treatment, but a patient deceased because of a urinary tract infection developing a colovesical fistula. This event occurred in 2nd cycle, and the patients did not have neutropenia at the beginning of 2nd cycle and during the 2nd cycle of treatment.

**Table 3:** Comparison of clinical events during the 4 cycles of pomalidomide treatment according to ANC increase at day 8 of 1st cycle of pomalidomide treatment.

Event	ANC increase more than 2x10^9^/L (n=9)	ANC increase less than 2x10^9^/L (n=24)	*p*-value
Clinical characteristics			
Age, median (range) Regimen, n (%) Pd PCd Prior line of therapy, median (range) Baseline neutropenia Gr1-4 Gr3-4	76 (67-83) 2 (22.2) 7 (77.8) 2 (2-3) 4 (44.4) 1 (11.1)	74 (56-85) 3 (12.5) 21 (87.5) 2 (2-6) 14 (58.3) 3 (12.5)	0.353 0.597 0.374 0.697 1.000
Adverse events			
Overall (Gr1-4) neutropenia, n (%)	1 (11.1)	18 (75.0)	0.002
Gr3 neutropenia, n (%) Gr4 neutropenia, n (%)	1 (11.1) 0 (0.0)	6 (25.0) 10 (41.7)	0.642 0.032
Duration of Gr3-4 neutropenia (days), median (range)	23 (19-28)	10 (1-34)	0.189
Overall (Gr1-4) Febrile neutropenia, n (%) Gr3 Febrile neutropenia, n (%) Gr4 Febrile neutropenia, n (%)	0 (0.0) 0 (0.0) 0 (0.0)	4 (16.7) 1 (4.2) 3 (12.5)	0.555 1.000 0.545
Overall (Gr1-4) Infection, n (%) Gr3 infection, n (%) Gr4 infection, n (%)	2 (22.2) 0 (0.0) 1 (11.1)	5 (20.8) 3 (12.5) 0 (0.0)	1.000 0.555 0.273
Dose adjustment (dose reduction or treatment interruption)			
Pomalidomide, n (%)	1 (12.5)	11 (45.8)	0.204
Cyclophosphamide, n (%)	3 (42.9%)	9 (42.9)	1.000
Dexamethasone, n (%)	4 (50.0)	5 (20.8)	0.152
Treatment delay > 2days, n (%)	3 (33.3)	14 (60.9)	0.243

Adverse events of pegfilgrastim are described in **Table 4**. The most common adverse event of pegfilgrastim was fatigue. Myalgia, weakness, nausea, vomiting, bone pain, febrile sense, and tremor were followed. All the adverse events were grade 1 or 2 and no medical intervention was needed.

**Table 4:** Adverse events of pegylated G-CSF.

Event	N=33
Fatigue	
Gr 1/2 Gr 3/4	3 (9.1)/2 (6.1) 0 (0.0)/0 (0.0)
Mylagia	
Gr 1/2 Gr 3/4	1 (3.0)/2 (6.1) 0 (0.0)/0 (0.0)
Weakness	
Gr 1/2 Gr 3/4	3 (9.1)/0 (0.0) 0 (0.0)/0 (0.0)
Nausea/Vomiting	
Gr 1/2 Gr 3/4	3 (9.1)/0 (0.0) 0 (0.0)/0 (0.0)
Bone pain	
Gr 1/2 Gr 3/4	0 (0.0)/2 (6.1) 0 (0.0)/0 (0.0)
Febrile sense	
Gr 1/2 Gr 3/4	0 (0.0)/1 (3.0) 0 (0.0)/0 (0.0)
Hand tremor	
Gr 1/2 Gr 3/4	1 (3.0)/0 (0.0) 0 (0.0)/0 (0.0)
Insomnia	
Gr 1/2 Gr 3/4	0 (0.0)/0 (0.0) 0 (0.0)/0 (0.0)
Headache	
Gr 1/2 Gr 3/4	0 (0.0)/0 (0.0) 0 (0.0)/0 (0.0)

### Response to treatment and survival outcome

Overall, 14 patients (24.4%) did not complete 4 cycles of pomalidomide treatment. The main reason for discontinuation of therapy was disease progression (11 patients, 78.6%) and adverse event (3 patients, 21.4%). All cases of treatment discontinuation due to adverse events were associated with infection. Dose reduction or treatment interruption of pomalidomide, cyclophosphamide, or dexamethasone occurred in 12/33 patients (36.4%), 12/28 patients (42.9%), and 9/33 (27.3%) respectively. The episodes of treatment delay of more than 2 days occurred in 17 patients (51.5%). The best response to treatment during 4 cycles of pomalidomide treatment was summarized in **Supplementary Table 2**. ORR was 54.5% and complete response (CR) was observed in 3% of the patients. ORR was 57.1% in patients receiving PCd and 40.0% in patients receiving Pd. Median PFS was 7.8 months (95% CI 7.085-8.582) in PCD-treated patients and 4.0 months (95% CI 0.961-6.973) in PD-treated patients (*p*=0.409). Median OS was 9.9 months (95% CI 6.549-13.184) in PCD-treated patients and 23.1 months (95% CI 9.612-36.522) in PD-treated patients, respectively (*p*=0.414).

### Peripheral blood mononuclear cell analysis

To assess the effect of pegylated G-CSF on the function of T lymphocytes, ex vivo proliferative response to polyclonal T lymphocyte mitogens of peripheral blood mononuclear cells (PBMC) from the patients was examined. Serial follow-up and analysis of peripheral blood mononuclear cell and cytokine analysis were available in 14 and 21 patients, respectively. The effector T-cell population and the regulatory T-cell population did not show significant change after the administration of pegylated G-CSF (**Supplementary Table 3**). Interferon-gamma (IFN-γ), IL12p70, and TGF-β analyses using enzyme-linked immunosorbent assay (ELISA) showed no significant change through the treatment periods (**Supplementary Table 4**).

## Discussion

This study evaluated the prophylactic effect of pegfilgrastim in decreasing the rate of severe neutropenia and febrile neutropenia in 33 patients with RRMM who were treated with PCd or Pd. All patients were relapsed or refractory status after more than two previous treatments. The incidence of grade 3-4 neutropenia and grade 3-4 febrile neutropenia was 53.6% and 12.1%, respectively. Grade 3-4 infection occurred in 15.2% of patients, but only 3 cases of infection were associated with neutropenia. All of the adverse reactions associated with pegfilgrastim were grade 1-2 and well tolerated.

Severe neutropenia during pomalidomide-based treatment was reported to be about 33-53% according to the previous prospective phase 2 trials (2, 3, 12). In a phase 2 study of PCd at first relapse after treatment with lenalidomide-bortezomib-dexamethasone in transplant-eligible patients, grade 3-4 neutropenia occurred in 51/97 patients (52.6%) and grade 3-4 infection occurred in 6/97 patients (6.2%). The median age of the included patients was 62 years and toxicity profiles were tolerable (12). In another phase 1/2 study of PCd in 69 patients, grade 3-4 neutropenia and infection occurred in 33.3% and 7.2% of patients, and 2 patients died of infection. These studies include transplant-eligible patients or relatively fit patients whose median age of less than 70 years and the results may be different from treatment outcomes of real-world data. The incidence of grade 3 or more neutropenia and infection in the present study seems to be higher than that of the previous prospective clinical trials. However, the patients included in our study were elderly, whose median age was 75 years, and 51.5% of patients had any grade of cytopenia at baseline. All patients received prior treatment including both bortezomib and lenalidomide before the study enrollment according to the Korean reimbursement guideline. Therefore, this prospective study reflects the real-world clinical outcome of MM patients and indeed, the incidence of grade 3 or 4 neutropenia and infection is considered low compared to the previous real-world data about PCd, in which grade 3-4 neutropenia of 97.4% and grade 3-4 infection of 62.2% were reported (4).

The major causes of cytopenia in MM patients are related to the effects of past chemotherapy and disease progression. Therefore, more patients have low blood cell count as treatment progresses. Neutropenia before starting chemotherapy is the primary risk factor for the development of neutropenic complications during and after treatment (13, 14). Pegfilgrastim has been studied in RRMM patients in several studies. In a prospective phase II trial about the bendamustine-lenalidomide-dexamethasone combination in RRMM, pegfilgrastim was administered to 68% of patients as primary prophylaxis. Although severe neutropenia occurred in 74% of all patients in that study, febrile neutropenia occurred in only 2 patients, and more patients administered pegfilgrastim received a completed planned schedule of treatment (15). In a phase 1/2 trial of 190 RRMM patients receiving lenalidomide, adriamycin, and dexamethasone (RAD) treatment, grade 3-4 neutropenia rate was 48.0% and grade 3-4 infection occurred in 10.5% of the patients. Although there was no control group to compare the incidence of neutropenia, the use of pegfilgrastim was suggested to increase the maximum tolerated dose level (16). In two retrospective studies about RRMM, pegfilgrastim showed superior efficacy in preventing prolonged neutropenia in terms of duration of neutropenia, and febrile neutropenia-related hospitalization periods than filgrastim (10, 17). In the present study, seventeen patients (51.5%) had any grade of neutropenia before starting pomalidomide-based treatment and the incidence of grade 3-4 neutropenia was higher than those without neutropenia at baseline, although statistically not significant. Although the incidence of grade 4 neutropenia was slightly higher in patients with baseline neutropenia, the incidence of grade 3 neutropenia or grade 3 or 4 febrile neutropenia did not show a difference according to baseline neutropenia. These results suggest that pegfilgrastim has a prophylactic role for relapsed refractory MM patients with or without baseline neutropenia.

Dynamic change of ANC appears different in healthy patients and chemotherapy-treated patients. In healthy patients, ANC reaches the maximum value around 4-6 days after administration of pegfilgrastim (18). In chemotherapy-treated patients, patients have the lowest ANC at day 7 and experience ANC recovery after day 7 (19). The dynamic change of ANC responding to pegfilgrastim in MM patients treated with immunomodulating drugs or proteasome inhibitors is different from conventional cytotoxic chemotherapy and there is no study about pharmacodynamic and pharmacokinetic analysis of pegfilgrastim in novel agent treatment of MM. Studies evaluating the effectiveness of pegfilgrastim in patients receiving IMiDs are rare, and the optimal timing of pegfilgrastim administration in the treatment of IMiDs remains unknown. The median onset of neutropenia during pomalidomide treatment has not been reported. The median time for neutrophil recovery after pegfilgrastim administration was 9 days. Pegfilgrastim is known to be cleared through a neutrophil-mediated mechanism, resulting in sustained serum concentrations of pegfilgrastim during the period of neutropenia. Therefore, we decided to administer pegfilgrastim on Day 1 of each treatment cycle (20). Interestingly, the patients with markedly increased ANC of more than 2 x 10^9^/L compared to baseline ANC after 7 days of pegfilgrastim at 1st cycle of treatment showed a significantly lower incidence of grade 3 or more neutropenia. As shown in **Figure 1**, the incidence of neutropenia and febrile neutropenia does not increase as the treatment cycle progresses and it means that the patients with neutropenia in the first cycle have repeated neutropenia during further treatment cycles. And there may be a biomarker predicting the effect of pegfilgrastim for preventing neutropenia. Although it is difficult to draw conclusions in this small-sized study, it is estimated that a good response to pegfilgrastim which is represented as ANC increase at day 8 of pegfilgrastim can reduce the incidence of grade 3 or more neutropenia regardless of baseline neutropenia.

In this study, serial peripheral blood samples were obtained during treatment, and immunologic changes according to the administration of pegfilgrastim were analyzed. A previous study reported that G-CSF induces MDSCs and influences tumor microenvironments in cancer (21–23). Results from experimental models, *in vitro* studies, and clinical data indicate that granulocyte colony-stimulating factor (G-CSF) stimulation can alter T-cell function and induces Th2 immune responses. Following *in vivo* G-CSF stimulation, both human and murine T cells have shown reduced cytotoxic activity and diminished proliferative responses upon *in vitro* stimulation (24–26). These results suggested that G-CSF or pegylated G-CSF treatment may have an immunologic adverse effect on cancer, and it raises a concern about promoting the disease progression of myeloma. Although there are only limited data about PBMC and cytokine analysis, there was no significant change in effector T-cell and regulatory T-cell population in this study. And there was no consistent increase of TGF-β or decrease of IL-12 and IFN-γ. This result suggests that pegylated G-CSF can be safely used for myeloma patients without concern about possible disease progression induced by G-CSF.

In conclusion, this study prospectively evaluated the safety and efficacy of pegfilgrastim as a primary prophylaxis of febrile neutropenia and infection among patients with RRMM who were treated with pomalidomide-based regimens. During the 4 cycles of treatment, any grade of neutropenia occurred in 19 patients (57.6%) and grade 3 or more neutropenia occurred in 17 patients (51.5%). Four patients (12.1%) experienced grade 3 or more febrile neutropenia. The patients with markedly increased ANC of more than 2 x 10^9^/L compared to baseline ANC after 7 days of pegfilgrastim at 1st cycle of treatment showed a significantly lower incidence of grade 3 or more neutropenia. It is challenging to conclude definitely that this study, using pegfilgrastim, clearly had an effect in reducing the frequency of severe neutropenia incidence when compared to previously published prospective clinical trials. However, considering that the patients included in this study were elderly and had baseline neutropenia, a population not typically included in general prospective studies, the low incidence of grade 3-4 febrile neutropenia and infection in this study holds significance. Further investigations are needed to assess the efficacy of pegfilgrastim prophylaxis in RRMM patients treated with pomalidomide in a randomized study.

## Data availability statement

The data may be obtained from the corresponding authors onreasonable request.

## Ethics statement

This study was approved by the institutional ethics committees of all participating institutions ans was conducted in accordance with the Declaration of Helsinki. Written informed consents were obtained from all participating patients.

## Author contributions

S-HJ, JK, and J-JL designed the study; G-YS and S-HJ prepared the manuscript; JM, DK, MK, HK, Y-CM, WL, YD, and JL critically reviewed the manuscript. All authors contributed to the article and approved the submitted version.
