# Supplementary material for: Open-labeled, multicenter phase II study of prophylactic administration of pegylated granulocyte colony-stimulating factor in relapsed or refractory multiple myeloma who received pomalidomide-based regimens (KMM170)
Source: Front Oncol. 2023 Oct 25;13:1209110. doi: 10.3389/fonc.2023.1209110 (PMC10642200; doi:10.3389/fonc.2023.1209110)
Supplement: Supplementary file 1 [file Table_1.docx]

**Supplementary materials**

**Open-labeled, multicenter phase II study of prophylactic administration of pegylated granulocyte colony-stimulating factor in relapsed or refractory multiple myeloma who received pomalidomide-based regimens (KMM170)**

Ga-Young Song^1†^, Sung-Hoon Jung^1†^, Joon Ho Moon^2^, Dajung Kim^3^, Min Kyoung Kim^4^, Hyo Jung Kim^5^, Yeung-Chul Mun^6^, Won-Sik Lee^7^, Young Rok Do^8^, Jae Hoon Lee^9^, Je-Jung Lee^1^, Jin Seok Kim^10^; the Korean Multiple Myeloma Working Party

^1^Chonnam National University Hwasun Hospital and Chonnam National University Medical School, Hwasun, Jeollanamdo, Republic of Korea; ^2^Kyungpook National University Hospital, School of Medicine, Kyungpook National University, Daegu, Republic of Korea; ^3^Kosin University Gospel Hospital, Busan, Republic of Korea; ^4^Yeungnam University Medical Center, Daegu, Republic of Korea; ^5^Hallym University Sacred Heart Hospital, Anyang, Republic of Korea; ^6^Ewha Womans University School of Medicine, Seoul, Republic of Korea; ^7^Busan Paik Hospital, Busan, Korea;^8^Keimyung University, School of Medicine, Keimyung University Hospital, Daegu, Republic of Korea; ^9^Gachon University Gil Medical Center, Incheon, Republic of Korea; ^10^Severance Hospital, Yonsei University College of Medicine, Seoul, Republic of Korea;

†These authors have contributed equally to this work and share first authorship

**Running Title:** pegfilgrastim for prophylaxis of febrile neutropenia in multiple myeloma

**Corresponding author:**

**Je-Jung Lee, M.D., Ph.D.,** Department of Hematology-Oncology, Chonnam National University Hwasun Hospital and Chonnam National University Medical School, 322 Seoyangro, Hwasun, Jeollanamdo, 58128, Republic of Korea; Tel: 82-61-379-7638; Fax: 82-61-379-7628; E-mail: drjejung@chonnam.ac.kr

**Jin Seok Kim, M.D., Ph.D.,** Division of Hematology, Department of Internal Medicine, Severance Hospital, Yonsei University College of Medicine, 50-1 Yonsei-ro, Seodaemun-gu, Seoul 03722, Republic of Korea, Tel: +82-2-2228-1972, Fax: +82-2-393-6884, E-mail: HEMAKIM@yuhs.ac

**Supplementary Table 1. Comparison of clinical events during the 4 cycles of pomalidomide treatment according to baseline neutropenia before pomalidomide treatment.**

|  | Patients with baseline  neutropenia (n=17) | Patients without baseline  neutropenia (n=16) | *p*-value |
| --- | --- | --- | --- |
| Overall (Gr 1-4) neutropenia, n (%)  Gr 3 neutropenia, n (%)  Gr 4 neutropenia, n (%) | 12 (70.6%)  4 (23.5%)  6 (35.3%) | 7 (43.8%)  6 (37.5%)  1 (6.3%) | 0.166  0.465  0.085 |
| Overall (Gr 1-4) infection, n (%) | 3 (17.6%) | 5 (31.3%) | 0.438 |
| Gr 3 infection, n (%)  Gr 4 infection, n (%) | 2 (11.8%)  0 (0.0%) | 2 (12.5%)  1 (6.3%) | 1.000  0.485 |
| Overall (gr 1-4) febrile  Neutropenia, n (%)  Gr 3 febrile neutropenia, n (%)  Gr 4 febrile neutropenia, n (%) | 3 (17.6%)  0 (0.0%)  3 (17.6%) | 1 (6.3%)  1 (6.3%)  0 (0.0%) | 0.601  0.485  0.227 |
| Number of patients whose ANC  increase more than 2ⅹ10^9^/L at  day 8 of 1st cycle, n (%) | 5 (29.4%) | 8 (50.0%) | 0.296 |
| ANC increase at day 8 of  1^st^ cycle, median (range) | 1100 (70-7990) | 2440 (140-6530) | 0.345 |

**Supplementary table 2. Best treatment response during 4 cycles of pomalidomide treatment**

|  | Total (N=33) | PCd (n=28) | Pd (n=5) |
| --- | --- | --- | --- |
| CR  VGPR  PR  SD  PD  ORR (≥PR) | 1 (3.0)  7 (21.2)  10 (30.3)  12 (36.4)  3 (9.1)  18 (54.5) | 1 (3.6)  7 (25.0)  8 (28.6)  10 (35.7)  2 (7.1)  16 (57.1) | 0 (0.0)  0 (0.0)  2 (40.0)  2 (40.0)  1 (20.0)  2 (40.0) |

N, number; CR, complete response; VGPR, very good partial response; PR, partial response; SD, stable disease; PD, progressive disease

**Supplementary Table 3. Effector T-cell and regulatory T-cell populations in PBMC cell fractions according to pegylated G-CSF administration during 4 cycles of pomalidomide treatment**

|  | C1D1 | C1D8 | C2D1 | C3D1 | C4D1 |
| --- | --- | --- | --- | --- | --- |
| CD3^+^ T-cell | 28.6  (1.0-90.5) | 25.3  (2.0-98.0) | 49.6  (0.5-85.0) | 79.9  (78.5-81.3) | 38.4  (11.0-92.0) |
| CD4^+^ T-cell  (CD3^+^CD4^+^) | 24.2  (4.0-54.0) | 30.9  (9.4-57.9) | 27.2  (8.0-45.0) | 29.5  (17.8-41.2) | 22.9  (12.8-44.3) |
| CD8^+^ T-cell  (CD3^+^CD8^+^) | 44.9  (27.1-83.0) | 43.6  (22.7-63.6) | 47.1  (20.3-88.0) | 41.3  (23.5-59.1) | 47.8  (22.7-64.4) |
| NK cell  (CD3^-^CD56^+^) | 50.0  (1.0-96.0) | 46.8  (3.0-94.2) | 71.3  (1.0-93.0) | 46.0  (11.0-81.0) | 57.4  (19.6-90.0) |
| EM T-cell  (CD3^+^CD62L^-^CD45RA^-^) | 37.0  (21.0-75.0) | 45.0  (20.6-74.6) | 27.1  (10.0-66.0) | 41.7  (40.0-43.3) | 43.3  (8.5-60.3) |
| CM T-cell  (CD3^+^CD62L^+^CD45RA^-^) | 12.0  (4.9-37.0) | 15.4  (2.0-35.1) | 12.3  (3.9-34.0) | 11.7  (11.3-12.0) | 6.3  (4.0-35.0) |
| Naïve T-cell  (CD3^+^CD62L^+^CD45RA^+^) | 10.7  (3.6-47.7) | 7.3  (1.2-52.0) | 18.7  (6.9-39.0) | 8.0  (2.9-13.0) | 15.5  (2.1-56.7) |
| Terminally differentiated T-cell  (CD3^+^CD62L^-^CD45RA^+^) | 27.2  (1.0-61.2) | 22.2  (14.3-60.7) | 25.5  (13.0-49.0) | 38.8  (35.6-42.0) | 21.8  (7.2-64.0) |
| Regulatory T-cell  (CD4^+^CD25^+^FoxP3^+^) | 3.4  (2.3-13.7) | 4.2  (1.2-12.0) | 4.7  (2.4-6.8) | 10.4  (3.2-17.5) | 4.2  (2.2-9.7) |

**Supplementary Table 4. Interferon-gamma (IFN-γ), interleukin (IL)-12p70, and tumor growth factor-beta (TGF-β) analyses using enzyme-linked immunosorbent assay (ELISA)** **according to pegylated G-CSF administration during 4 cycles of pomalidomide treatment.**

|  | C1D1 | C1D8 | C2D1 | C3D1 | C4D1 |
| --- | --- | --- | --- | --- | --- |
| IFN-γ | 18.58  (4.62-661.73) | 20.31  (2.19-1411.85) | 49.96  (8.77-162.58) | 23.31  (8.54-97.85) | 16.38  (4.04-53.31) |
| IL12p70 | 55.5  (17.00-166.50) | 73.00  (14.00-355.00) | 70.50  (7.50-198.00) | 49.00  (16.50-221.00) | 41.00  (8.00-103.50) |
| TGF-β | 10,473.8  (720.0-21667.5) | 9,350.6  (82.5-21,986.3) | 12,168.8  (5,985.0-32,216.3) | 13,436.3  (8,400.0-27,191.3) | 11,593.1  (6,120.0-23,268.8) |
